# Automated compound classification using a chemical ontology

**DOI:** 10.1186/1758-2946-4-40

**Published:** 2012-12-29

**Authors:** Claudia Bobach, Timo Böhme, Ulf Laube, Anett Püschel, Lutz Weber

**Affiliations:** 1 OntoChem GmbH, Heinrich-Damerow-Str. 4, Halle/Saale, D-06120, Germany

## Abstract

**Background:**

Classification of chemical compounds into compound classes by using structure derived descriptors is a well-established method to aid the evaluation and abstraction of compound properties in chemical compound databases. MeSH and recently ChEBI are examples of chemical ontologies that provide a hierarchical classification of compounds into general compound classes of biological interest based on their structural as well as property or use features. In these ontologies, compounds have been assigned manually to their respective classes. However, with the ever increasing possibilities to extract new compounds from text documents using name-to-structure tools and considering the large number of compounds deposited in databases, automated and comprehensive chemical classification methods are needed to avoid the error prone and time consuming manual classification of compounds.

**Results:**

In the present work we implement principles and methods to construct a chemical ontology of classes that shall support the automated, high-quality compound classification in chemical databases or text documents. While SMARTS expressions have already been used to define chemical structure class concepts, in the present work we have extended the expressive power of such class definitions by expanding their structure-based reasoning logic. Thus, to achieve the required precision and granularity of chemical class definitions, sets of SMARTS class definitions are connected by OR and NOT logical operators. In addition, AND logic has been implemented to allow the concomitant use of flexible atom lists and stereochemistry definitions. The resulting chemical ontology is a multi-hierarchical taxonomy of concept nodes connected by directed, transitive relationships.

**Conclusions:**

A proposal for a rule based definition of chemical classes has been made that allows to define chemical compound classes more precisely than before. The proposed structure-based reasoning logic allows to translate chemistry expert knowledge into a computer interpretable form, preventing erroneous compound assignments and allowing automatic compound classification. The automated assignment of compounds in databases, compound structure files or text documents to their related ontology classes is possible through the integration with a chemical structure search engine. As an application example, the annotation of chemical structure files with a prototypic ontology is demonstrated.

## Background

Ontologies are formal representations of knowledge concepts about objects and their relations in a specific domain [1]. While biology related ontologies have made a great impact on knowledge and data mining in life sciences, chemical ontologies that can be used for semantic data mining are just at the dawn of their development. Searching for chemical compound classes and related data has traditionally been the area of chemistry experts, using chemical structure databases and searching for individual structures, similar structures or sub-structures with specialized chemistry search engines. Chemical ontologies try to make this chemistry knowledge available to a broader community of scientists [2], allowing to classify and retrieve data on compounds and their classes more easily also by non-chemists. In addition, chemical ontologies may enable new ways of knowledge discovery for example by extracting relationships between compound classes and related data from other domains, which are traditionally known as structure-activity relationships (SAR) or structure–property relationships (SPR). Thus, to provide an answer to the query “Which diseases can be treated with monoterpenes?” requires the integration of a disease and chemistry ontology, supplemented by a proper relationship detection method. While chemical ontologies may serve very different purposes in data mining, the current paper specifically aims at the implementation of a suitable chemical ontology that enables the automated annotation of compounds to compound classes. These annotations could then be used for the annotation of text documents and subsequent extraction of compound related SAR or SPR facts and knowledge by data mining methods which are beyond the scope of this present work.

Chemists, similar to biologists building taxonomies of living species, were early on classifying compounds into groups based on their various properties. Starting initially with taste and smell derived properties like “sweet”, “salty” and “sour”, the knowledge of sophisticated structure based classifications has become the core expertise of chemists. Thus, a range of software tools have been developed that allow to correlate the structure of a chemical scaffold and biological activities for example by utilizing chemical structure based hierarchical ontologies.

In the past few decades, chemical ontologies have been proposed and implemented to index text documents for domain specific search engines. One of the first examples was the MeSH (Medical Subject Heading) controlled vocabulary thesaurus [3] that is used for indexing articles in PubMed [4]. The D sub-tree of the MeSH 2012 vocabulary contains chemical classes, individual compounds and biological concepts which are classified using a Dewey decimal classification system. In total, the tree contains 9,096 compound and compound class nodes with 68,822 synonyms (April 2012) that are used for the annotation of the abstract text. Compound classes do not include chemical structure definitions that would allow for an automated classification and the MeSH classification hierarchy has been built manually. A range of other chemical ontologies have been proposed to represent certain sub-aspects of chemistry, specific compounds or chemical classes. An example for ontology definitions specifically for lipids is LIPIDMAPS [5], glycanes are described in the Glycomics Ontology (GlycO) [6]. The currently most comprehensive open source chemical ontology of compounds and compound classes is ChEBI (Chemical Entities of Biological Interest) ontology [7,8]. In total, ChEBI (version 88, February 2012) contains 30,944 chemical compound and class nodes with 183,608 synonyms that could be used for text mining. ChEBI also provides extensive links to other databases with compound information in the biomedical field. Similar to MeSH, the annotation of specific compounds to compound classes is performed manually. An interesting application of ChEBI is ARISTO [9] which provides assignments to ChEBI using a mass spectrum of compounds as input. Most recently, desiderata for automated structure-based classifications have been formulated, outlining also logical rules for chemical reasoning and their implementation in formal OWL expressions [10]. A general ontology for chemistry terms beyond compound classes has been introduced in the Chemical Information Ontology CHEMINF [11] and the integration of these ontologies into dedicated text processing engines has advanced significantly for example by the open source OSCAR4 [12] that can be used to annotate scientific text documents with chemical terms and classes.

To circumvent the labour intensive, error prone manual assignment of individual compounds to specific compound classes such as realized in MeSH or ChEBI, efforts have been made to automatically classify compounds through the structural definition of compound classes and the concomitant use of a structural search engine for executing the classification. For example, a compound will be assigned to be a member of a particular chemical class if its structure is a superstructure of the class definition – or in other words – it contains the structure definition of the respective chemical class as a substructure. The functional groups ontology FGO [13] and the Chemical Ontology (CO) [14] have proposed this method for the automated assignment/identification of functional groups in compounds, using 231 functional group definitions for substructure searching. Similarly, ChemEd has been proposed as an editor to assign functional groups automatically [15]. A recent example for both automated ontology construction as well as automated compound assignment using the resulting ontology is Scaffold Hunter by Novartis [16]. Here, chemical scaffolds are extracted automatically from compound structures by dissecting compounds according to predefined rules, for example cutting of non-cyclic substituents. A scaffold is then defined as a class and being a parent class of another scaffold if it is a sub-structure of this other scaffold, creating thereby automatically a comprehensive hierarchy of scaffolds. Subsequently, compounds are classified into their matching scaffold classes by automated chemical sub-structure searches. A disadvantage of this method is that only cyclic structures are considered as scaffolds and that important biologically relevant scaffold types are missing. Chem-BLAST [17] is a similar program developed by the National Institute of Standards and Technologies and uses its own scaffold extraction methods. The very recent example of this ontology development strategy is focussing to create a structure based ontology hierarchy in a semi-automated, “self-evolving” way and to automatically assign compounds to these classes [18]. In this interesting work, a manually assembled training set of molecules belonging to a particular compound class is used to automatically identify consensus substructures that will then serve as a structure-based definition of this particular class. The advantage of the above described structure-based ontology creation method is obviously its straightforward implementation as well as that final hierarchies are derived by algorithmic rules automatically.

However, the resulting hierarchies may not yet capture the full complexity of traditional compound classifications. Especially complex biologically relevant compounds may often be described by sets of multiple scaffold structures and for an individual compound such as for example tautomeric or stereoisomeric forms. Thus, a good example are the three different tautomeric forms of vitamin C – each of them being a correct description but in a given chemistry database typically only one structural form will be represented. Another example is glucose with its open chain and the two cyclic forms of D-glucose, the pyranose and the furanose form, requiring all three structural forms in the ontological definition of a chemical glucose class to allow glucose or glucose derivatives to be classified automatically from chemical structure files. Another very important aspect for the proper definition of compound classes is the notion of the absence of a particular chemical substructure. For example, the alkane compound class should NOT contain any other atom except carbon and hydrogen. To achieve this requirement, one has to add also structural definitions that shall not be part of compounds that belong to a specific class. This aspect is only poorly or not at all covered by automated methods such as described in [18].

The present article is therefore aiming to develop principles and methods for the construction of a chemical expert ontology of compound classes that shall allow to represent the high complexity of chemical classifications better than before and that could be used for automated classification of compounds in databases or text documents.

## Results

### Chemical terminology: compounds and compound classes

Chemical compound related named entity terms as they are used in biomedical documents or databases include not only compound names but also terms for general compound classes, chemical scaffolds, class derivatives, chemical substituents and functional groups - for information retrieval (IR) purposes it is important to identify, classify and separate the meaning of these various terms. Each of those concepts represent chemical ontology classes exhibiting specific structural particularities allowing to distinguish those general terms as discussed in the following sections.

**Figure 1:**
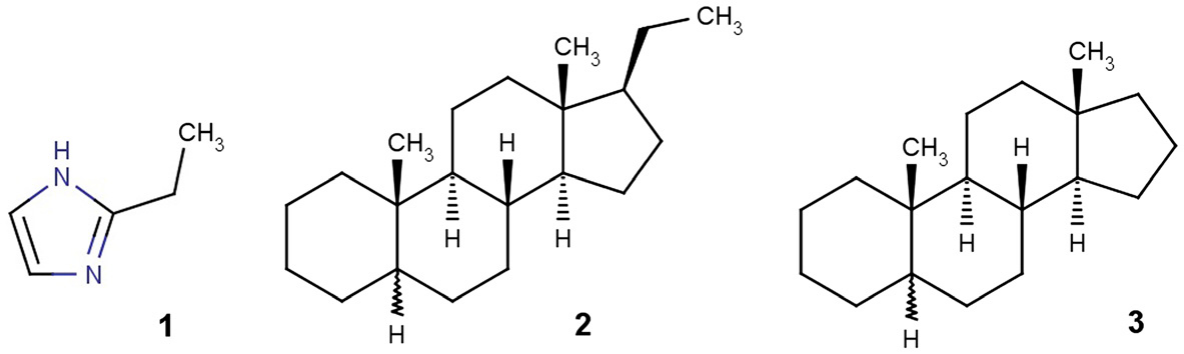
Structures of 2-ethyl-imidazole 1, pregnane 2 and androstane 3.

Chemists use a variety of expressions to create compound class terms from a specific compound name – for example “backbone”, “scaffold”, “derivative”, “compound class” are often used suffixes or “substituted” is a common prefix that generates a class term. Unfortunately, the meaning of different chemical class terms is often not defined precisely and their usage may differ significantly due to historic reasons and depending on the compound class. For example, 2-ethyl-imidazole **1** belongs without doubt to the class of compounds having an imidazole scaffold, backbone or being an imidazole derivative or substituted imidazole. In contrast, pregnane **2** illustrates a more complicated case – as in case of 2-ethyl-imidazole this compound could be considered a 17-ethyl-derivative of the androstane scaffold **3**. However, this would suggest a wrong compound classification as pregnanes are not considered to be androstane derivatives - although **2** contains androstane 3 as a substructure (Figure 1). This particular, structurally illogical naming convention goes back to the fundamentally different biological activities of specific compounds with a pregnane or androstane backbone, resulting in the perception that androstanes and pregnanes do not show a parent–child relation but are rather sibling concepts at the same hierarchical level. Thus, any expert chemical ontology will appreciate this knowledge and the androstane compound class structural definition needs to contain a definition that any androstane shall NOT contain a carbon substitution at the C-17 position.

The requirement that certain structural features shall not be present in a given compound class is not only limited to difficult natural product derived examples such as shown above but rather represents a general feature of chemical classes. For example, alkanes are compounds that only contain carbon and hydrogen atoms but not any other atom type. Similarly, saturated compounds shall not contain double bonds, inorganic compounds shall not contain carbon-carbon bonds etc. These few examples shall illustrate that proper definitions of chemical classes must contain rather complex structure definition sets. A further consequence is also that simple substructure searching, e.g. using the androstane scaffold as a substructure query, will lead to wrong classifications and should not be used for sophisticated ontological compound classification tasks. Instead, structural requirements need to be defined by sets of structural conditions to be present or absent - each of them triggering a structure query and having logical rules that handle the hit sets and give a final decision whether or not a specific compound or compound class will belong to the investigated chemistry ontology class.

### Definition of compounds

The International Union of Pure and Applied Chemistry (IUPAC) has developed a comprehensive terminology of general chemical terms and compound class definitions [19] that can provide a guidance for the development of an expert chemical ontology. For example, according to IUPAC a chemical compound is a pure chemical substance, consisting of two or more chemical elements with a fixed ratio of atoms, and having a unique and defined chemical structure. This requirement can be translated into a suitable structural representation using one or more connection tables (CT) of the same defined atoms. Connection tables can be represented by a variety of file formats such as SMILES, MOL, MOL2, MRV, CML or others.

**Figure 2:**
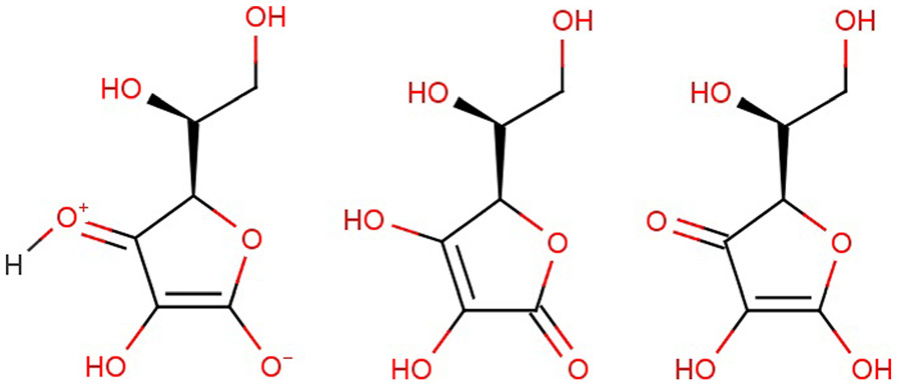
3 tautomeric forms of vitamin C.

In this context, it is interesting to consider the example of vitamin C for a precise definition of a compound and possible sets of structural definitions. Vitamin C can be described by a CT of non-hydrogen atoms bonded to each other in the same way, but the connection of vitamin C hydrogen atoms as well as the bond orders between non-hydrogen atoms may vary in the different tautomers of vitamin C (Figure 2). As the ratio of these tautomers depends on factors such as temperature, solvent, pH and others, we propose that all tautomers alone or in combination should be considered valid representations of the same chemical compound and resulting in a set of three structural definitions to describe the vitamin C class of compounds. As each of those structure definitions are valid representations of vitamin C, these definitions shall be connected by OR logic which means that a compound which satisfies only one of those structural definitions shall be a member of this chemical compound class.

**Chemistry ontology rule 1**: A chemical compound is a compound class having a fixed ratio of defined atoms and a chemical structure that can be expressed by one connection table of non-hydrogen atoms and one or more connection tables that include hydrogen atoms and bond orders as well, connected by OR logic.

According to this rule, compounds that occur as different microspecies for example as neutral or zwitter ionic species such as amino acids shall be represented by two or more connection tables as representations of the same compound. Rule 1 is also proving to be useful when considering that different CTs may be found in chemical databases for the same compound – especially if structures are not normalized by the same process before the structural representation is entering the database. Thus, irrespective which CT of the possible is actually found in the database, the correct class will be assigned to this data entry.

Rule 1 however does not capture for example polymers which are rather mixtures of compounds and which should be therefore treated differently. Also, one has to keep in mind that connection tables are based on the valence bound theory (VB) which is already a simplification of molecular reality. Thus, apart from known problems with metallo-organic compounds even some defined pure organic compounds cannot be represented well by VB-theory, for example, in certain pentalenes actually the connectivity between non-hydrogen atoms and their valency may change or is indefinable [20]. However, the proposed method allows for a straightforward annotation of compounds in databases that usually represent a particular compound only by one CT from the different possible. As the required different microspecies CTs could be generated from one starting structure by automated structure normalization methods it should be also possible to automatically generate the various structure definition sets for the chemical ontology and structure-to-name tools could automatically generate their corresponding names. Compounds that would not follow rule 1 are for example salts that could be described either in their ionic or covalent form with two CTs – for example using the SMILES [Na+].[Cl-] or [Na] [Cl] for sodium chloride. To capture these as one compound, normalization of structures is definitively required.

Stereoisomers pose another interesting problem for an expert chemical ontology – for example, the term “lactic acid” may refer to the naturally occurring D-(−)-lactic acid, also known as (R)-lactic acid, where the explicit stereochemistry specification has been omitted in the text. Alternatively, it may refer to the racemate rac-lactic acid which is a 50:50 mixture of (S)-lactic acid and (R)-lactic acid, or to 2-hydroxy-propanoic acid with unknown or undefined stereochemistry. Thus, in the ontology this stereochemistry situation may be represented by three child compound nodes C[C@@H](O)C(O)=O (R-lactic acid), C[C@H](O)C(O)=O (S-lactic acid) and C[C@@H](O)C(O)=O.C[C@H](O)C(O)=O (rac-lactic acid), connected to a parent compound node without stereochemistry information CC(O)C(=O)O.

In an other example, the defined stereoisomer D-glucose could be represented by a furanose, a pyranose and an open chain structure. Thus, D-glucose could be represented in an ontology concept that encompasses three related SMILES connected by OR logic to define this compound. Please note that in the example the stereochemistry at the C-1 atom is not defined.

[H]OC([H])([H])[C@@]([H])(O[H])[C@@]1([H])OC([H])(O[H])[C@]([H])(O[H])[C@@]1([H])O[H] OR

[H]OC([H])([H])[C@@]1([H])OC([H])(O[H])[C@]([H])(O[H])[C@@]([H])(O[H])[C@]1([H])O[H] OR

[H]OC([H])([H])[C@@]([H])(O[H])[C@@]([H])(O[H])[C@]([H])(O[H])[C@@]([H])(O[H])C([H])=O

Similar to the described stereochemistry and compound isomer problems, we may regard compounds with different isotopic substitutions either as synonyms of the isotopically undefined compound or, also depending on the purpose of the ontology, as child concepts of the same. In general, IUPAC has developed extensive guidelines for naming compounds that could serve as a prototype and guide for developing a chemical ontology [21].

### Representation of compound classes

Building on the above definition of compounds, compound classes could be regarded as collections of compounds with chemical structures expressed by different connection tables connected by OR logic. A suitable representation of compound classes has been given by David Weininger by logical expressions in the SMARTS notation [22] that allow to precisely define structural elements that shall or shall not be present in compounds belonging to an assigned compound class. For example, a definition of a primary amine can be given by a SMARTS expression [N;H2v3][#6;!$(C=[O,S,N,P])] which requires that two hydrogen atoms are connected to a three-valent nitrogen atom connected to two hydrogens and a carbon atom that shall not be bound to oxygen, sulfur, nitrogen or phosphorous with a double bond. To capture the whole complexity of chemical class definitions however it is not enough to use only one SMARTS expression. Using multiple SMARTS, almost any definition of a chemical compound class can be constructed if those are combined by logical AND, OR and NOT operations.

The following examples shall illustrate this method: Cycloalkanes are compounds that only contain saturated carbon and hydrogen atoms and at least one ring system. This definition can be represented by [#6R]@[#6R] AND NOT ([#6R]:[#6R] OR [#6R]#[#6R] OR [#6R]=[#6R] OR [#2,#3,#4,#5,#7,…,#104]) which shall mean that the complete expression and classification is only true if the studied compound matches all SMARTS criteria. While [#6R]@[#6R] stands for a structure that contains a ring bond between two carbon atoms, a “pure” cycloalkane should not contain any aromatic, double or triple bonds as well as any other elements except carbon and hydrogen. These logical expression sets need to be implemented with a suitable structure search engine that understands SMARTS expressions to automatically classify compounds in databases, such as the ChemAxon [23] or Daylight [24] software tools.

However, using SMARTS raises several problems that require specific attention in a chemical ontology. For example, if atom lists shall be used in SMARTS description of a molecule class, the correct assignment of R or S tetrahedral stereochemistry or the E/Z double bond stereochemistry is not possible as it may change when using atoms from the list with different priorities. This problem can be circumvented by using two separated SMARTS expressions connected by an AND logic, one without atom lists defining the correct stereochemistry and one without stereochemistry but with all atom lists.

Another problem is to prevent a carbon-substitution at a specific carbon atom (such as in androstane 3 at atom C-17). All possible valences have to be defined by a non-carbon-list and also double bonds (e.g. a possible keto group substitution at this atom) have to be considered.

**Chemistry ontology rule 2**: A compound class definition may be built from logically connected SMARTS criteria. All sets of allowed or not allowed SMARTS are connected with OR logic. Two SMARTS expressions that shall be valid at the same time shall be connected with an AND logic, such as SMARTS containing stereochemistry information and SMARTS containing atom lists. The complete set of allowed and prohibited SMARTS expressions are finally connected with an AND NOT logic: (SMART-1 OR SMART-2 OR …) AND NOT (SMART-x OR SMART-(x+1) OR …).

As already mentioned before, many compound classes (e.g. “monoterpenes” or “lipids”) are not characterized by one common substructure criterion. However, it might be easier to define such classes through their children or descendants that have defined structural definitions - an expert will need to decide which classes shall be added to the parent term by assigning descendants that have SMARTS definition sets.

Considering the typical use of chemical class terms in scientific literature, it appears to us that it also makes sense to distinguish between two principal compound class types:

Compound classes with a narrow structure definition (as above shown for cycloalkanes). This will be the case for classes that are traditionally interpreted according to their historic biological, biophysical or other derivational characterization such as for example monosaccharides, lipids, steroids or monoterpenes. Often, the terminology for these compound classes uses the plural form “s”.

Compound classes with a broad structure definition. These could be derivatives or substituted compound classes that have been chemically modified to also contain substitutions that are typically not found in compounds assigned with the narrow definition. For example, compounds such as per-O-benzyl-glucose will not be found in nature, but may be synthesized chemically and could be assigned to the class “glucose derivatives”.

Further examples of broad and narrow definitions of related classes are:

The narrow class “carboxylic acids” class will require the presence of a COOH group, while the broad “carboxylic acid derivatives” class could contain carboxylic acid esters, chlorides or even amidines but in any case compounds where the COOH group is modified. Clearly, the classes “carboxylic acids” and “carboxylic acid derivatives” are on the same hierarchical level.

Similarly, “vitamins” means a group of specific and defined compounds, while “vitamin derivatives” are chemically modified vitamins and should not be considered vitamins in the narrow sense.

**Chemistry ontology rule 3**: Compound classes may be defined by narrow or broad structure definitions comprising one or multiple SMARTS definitions connected with AND, OR and NOT logic.

For an easier understanding, we propose that the name of the narrow class shall be a compound class name in plural form while the derived broader class shall contain the term “derivatives”.

The above definitions are of course arbitrary and the view on what is a narrow or broad class could vary significantly from chemist to chemist. For example, whether benzimidazole is considered a descendant of “imidazoles”, “imidazole derivatives” and “benzenes” or not will depend on the design principles used for constructing the ontology. In our opinion, fused ring systems such as “benzimidazoles” shall rather represent a distinct ontology class - in this case being a child of “bicyclic heterocylic ring systems”, rather than being a substituted imidazole. To prevent that benzimidazole is annotated as an imidazole, one may use SMARTS definitions that require each atom of the imidazole ring to be part of one ring system only.

### Relationships in chemical ontologies

An extensive review of possible relationships between chemical compounds has been proposed by J. Gordon [25]. ChEBI defines 10 different relationship types such as the commonly used *is_a* and *has_part* relationships, but also chemistry specific *is_conjugate_base_of*, *is_conjugate_acid_of*, *is_tautomer_of*, *has_parent_hydride* and *is_enantiomer_of* as defined by IUPAC rules. To enable a more seamless integration of the chemical ontology with simple search engines, we have used currently the *is_a* relationship only, providing the ontology in the form of a directed acyclic graph (DAG), also known as a taxonomy. Since the *is_a* relationship is transitive and directional, all properties of the parent class such as being a drug are also properties of the connected child compound classes. Transitivity of all concept properties is an important feature of the *is_a* relationship (if A → B AND B → C than it is also true that A → C).

A typical problem of manual assignments are redundant or missing links between ontology classes. Thus, manual ontology construction may lead to both over-assignments or missing assignments. Missing links will result in a reduced hit rate – for example the MeSH node “steroids” has not been linked as a child concept to the class of “terpenes” – searching with the query term “terpenes” in PubMed will therefore not return steroids as query results which might be expected by an expert.

Ontology editors have reasoning tools to discover such logical errors like discovering redundant links or cycle check routines. The latter may report an error if a chain of one or more links exist that make a term an ancestor of itself – which is not allowed in DAG type ontologies. The logical nature of structure based definitions allows to implement logical checks or reasoning that is specific for chemistry – for example checking if a compound SMILES satisfies all SMARTS definitions of its parent chemical classes in the hierarchical node chain. Chemical reasoning may also check which sub-structural parts of the compound are matched by the corresponding SMARTS of the compound class of interest, facilitating thereby the development of the ontology.

**Chemistry ontology rule 4**: When ontology classes are connected by an *is_a* relationship, the child node inherits all properties such as all parent compound structure SMARTS definitions connected with an AND logic.

A useful chemical ontology editor will implement this rule and will only assign compounds to a particular child class if all ancestor criteria are fulfilled, together with additional SMARTS properties of this class.

### Chemical Ontology

We have developed a prototypical chemical ontology using the proposed rules as described above for a structure based classification system. Particular attention has been given to natural products, e.g. steroids or sugars, but also to classes of heterocyclic compounds and compound classes that are of special interest for medicinal chemistry. In addition, property based classifications such as vitamins, flavours and fragrances, drugs and FEMA compounds have been defined through lists of specific compounds. Not described are polymers including large peptides, proteins and nucleic acid polymers as well as combinatorial libraries or mixtures. To capture the logic of these compound classes, SMARTS could be replaced by Daylights CHORTLES [26] or ChemAxon’s Markush enabled Marvin format [23] as class properties for automated assignment.

The prototypic chemical ontology was constructed in the “human readable” OBO format and contains 3800 compound classes, with a total of about 13,000 synonyms and with about 2800 classes being defined by SMARTS expressions. The depth of the DAG classification is 15 levels. Top level hierarchical nodes are:

Action and usage classification, containing biological agents, pharmacological agents, special agents and toxic agents

Derivational classification, such as natural products

Structural classification, such as compounds, elements, ions and radicals

The structural classification node classifies compounds into structure related groups. The next lower level of the compounds classification node contains the nodes: acyclic compounds, cyclic compounds, charged compounds, element compounds, hetero compounds, organic compounds and inorganic compounds.

**Figure 3:**
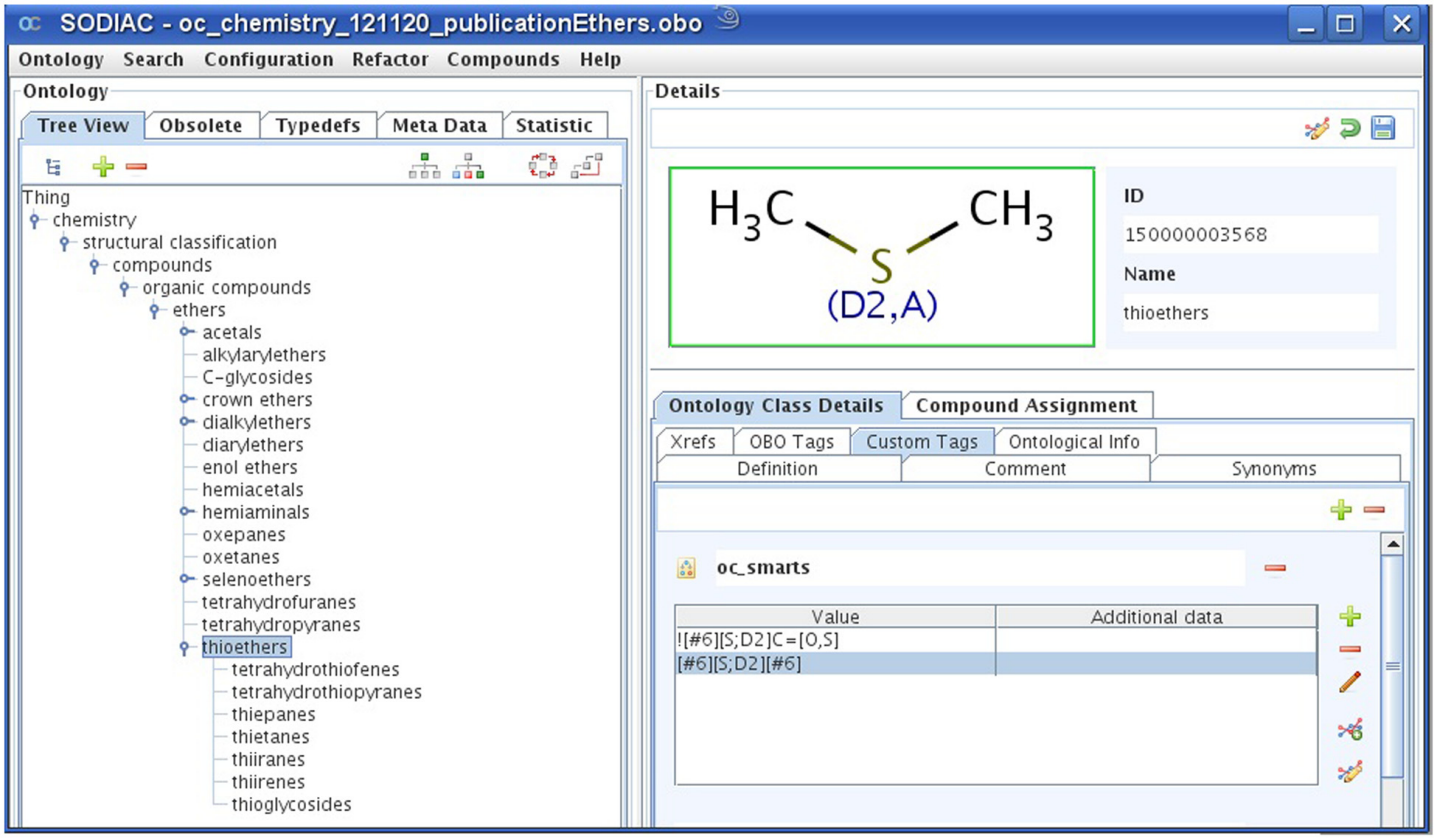
Subtree ontology A.obo describing functional groups, e.g. ethers.

**Figure 4:**
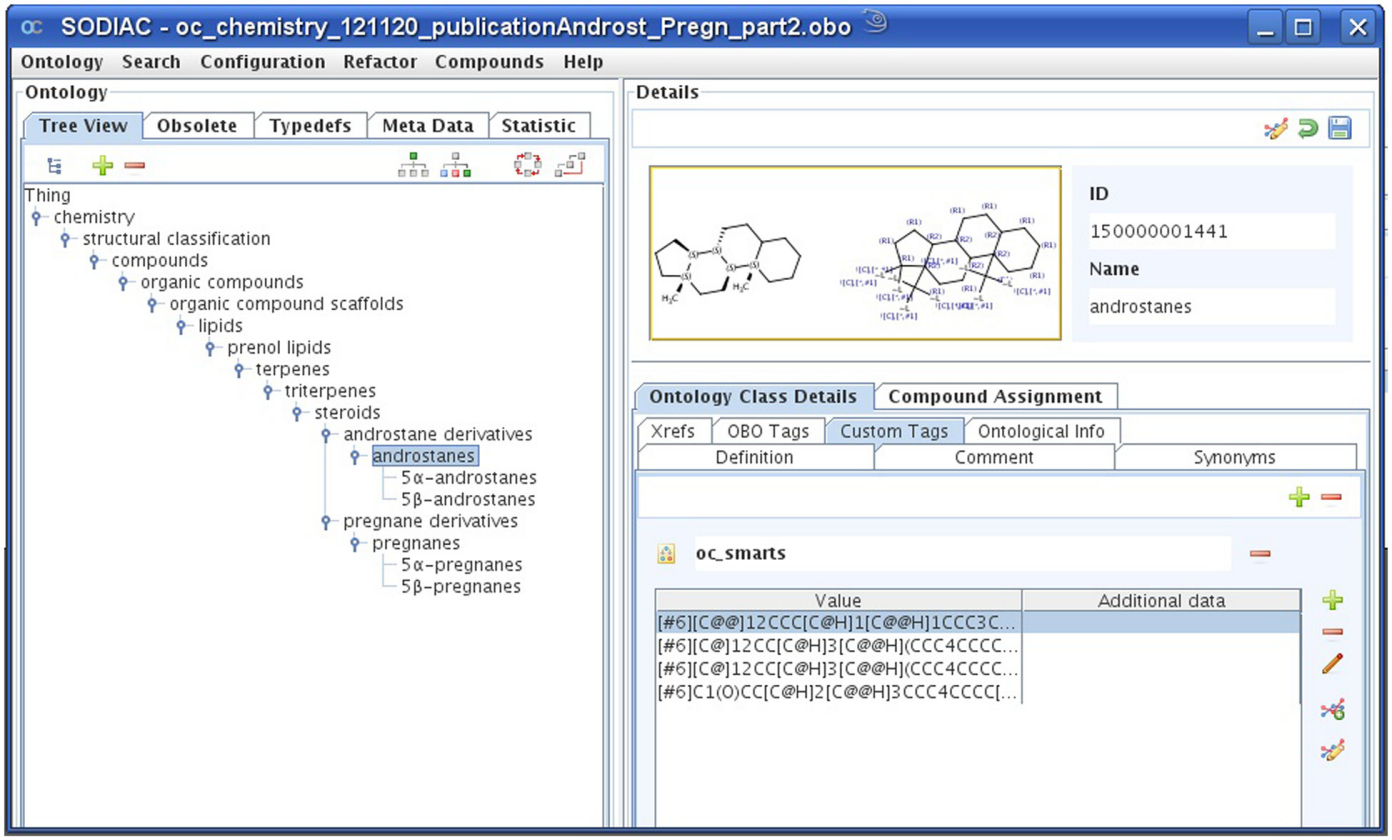
Ontology B.obo describing the androstane and pregnane subtree.

For the purpose of this article and for better clarity we have included two freely available subtrees of this ontology to demonstrate the outlined general construction principles of the generated chemical ontology. Ontology A (A.obo, see Figure 3 and Additional file 1: A.obo) is an example of the classification regarding functional groups such as ethers while ontology B (B.obo, see Figure 4 and Additional file 2: B.obo) represents the expert knowledge of more complex class definitions like androstanes and pregnanes. All other classes were removed from the complete ontology that are not relevant for the classification of ether or androstane and pregnane compounds.

### Automated assignment of compounds

Manual assignment of compounds to compound classes might be possible for some few thousand compounds but is impossible for large compound databases or structure data files. In addition, manual assignment is always prone to errors. We therefore used the constructed ontology described above to automatically assign compounds to their corresponding chemical classes.

All compounds were subject to structure evaluation with the ontology definitions and for each path the deepest matching ontology node was assigned as a parent of the respective compound. For testing the automated annotation of compounds, a chemical database of compounds was built, containing their chemical names and synonyms derived from public sources. On average, each PubChem compound was assigned to 15 parent compound classes with this procedure.

**Figure 5:**
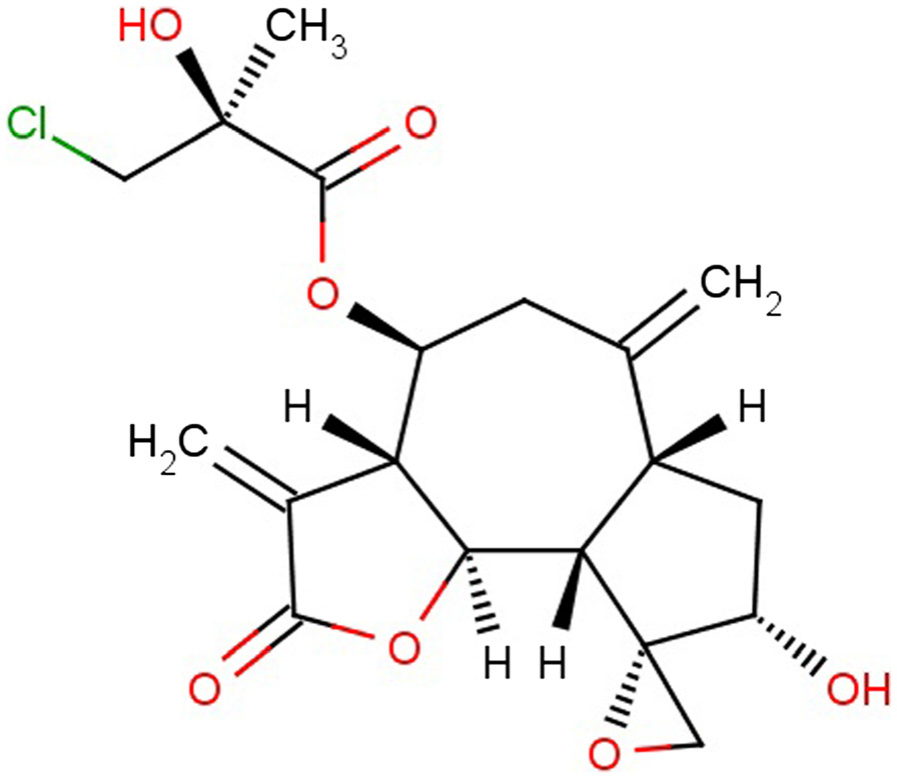
Structure of acroptilin.

For example, acroptilin (Figure 5) is put into 19 categories: alkene derivatives, alkyl chlorides, carboxylic acid esters, epoxides, lactones, secondary alcohols, tertiary alcohols, spiro-, hetero-, polycyclic-, chlorine-, carbon-, oxygen-compounds and natural product derivatives, bioavailable compounds, lead like molecule, Lipinski compounds, lipophilic compounds, low molecular weight compounds. In ChEBI, acroptilin has only two parents: azulenofuran and sesquiterpene lactone.

To illustrate the classification performance of this approach we assigned 198 compounds from [18] to the complete hierarchy of chemical classes. From this test set a compound was assigned directly to an average number of 8 classes. We determined an annotation precision for this test set of 95% (see supplied Additional file 3). The main reason for wrongly or missing annotated classes was due to an incorrect understanding of the chemical class “cycloarenes”. Cycloarenes are defined as polycyclic aromatic compounds in which the annelation of arene units form a macrocyclic structure. Erroneously the ontology class “cycloarenes” was considered as simply cyclic aromatic systems in our initial ontology.

**Figure 6:**
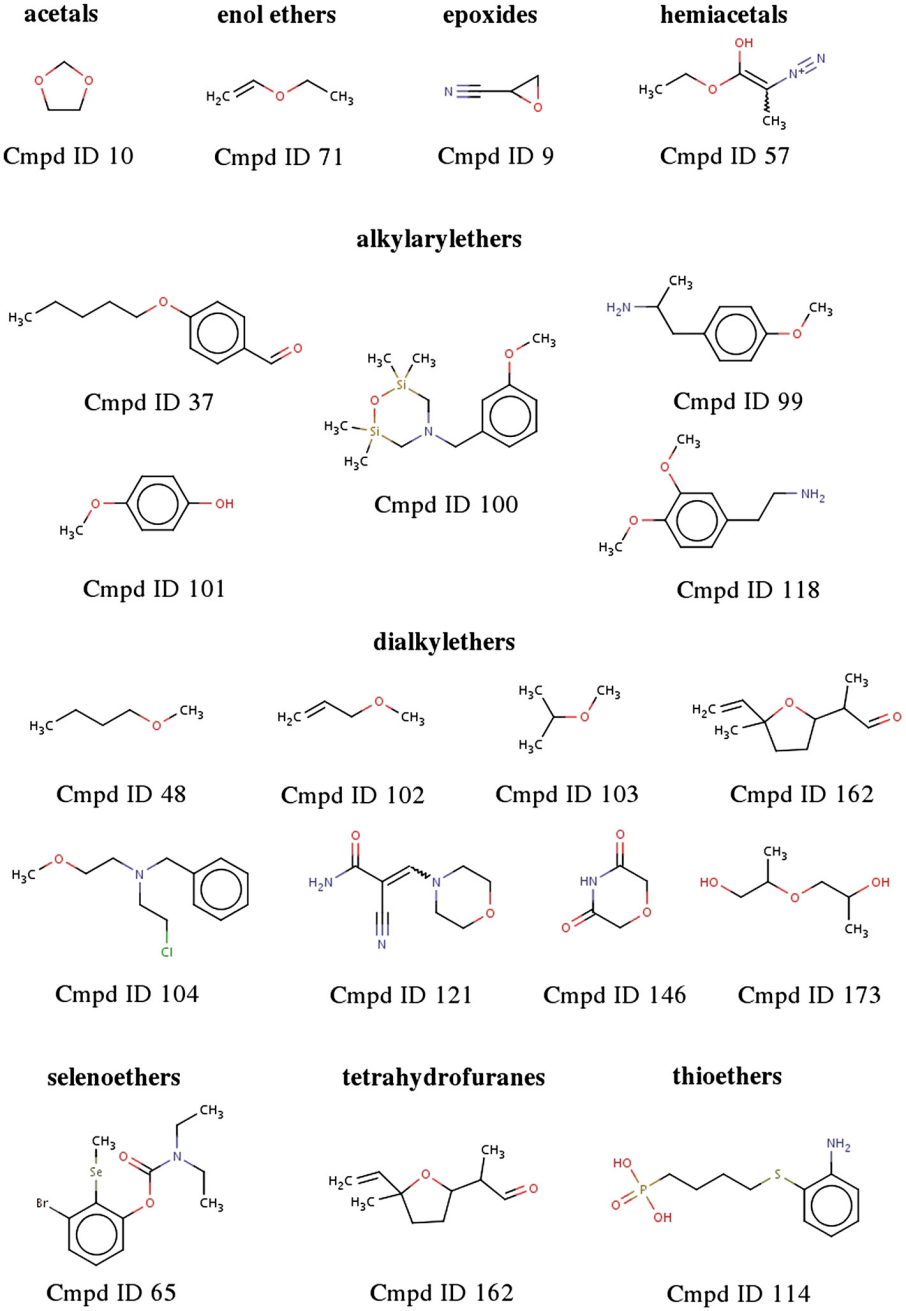
20 compounds from the 198 compound test set which were assigned to ether classes.

To describe the classification performance in more detail we also assigned this test set to the included parts of our complete hierarchy, ontology A and ontology B. For the ether class ontology A we could assign all ether group containing compounds to their corresponding ether classes. No false assignments and no missing assignments for the existing classes were recorded (precision 100%). The assigned compounds are shown in Figure 6.

We further evaluated our compound assignment performance using ontology B containing androstane and pregnane classes. For this purpose we assigned compounds from a 28,683,050 million compound database build from PubChem data source. Please note that for clarity, compounds were assigned to the deepest child node in the hierarchy only. The following frequencies have been obtained: androstanes – 502, 5α-androstanes – 1664, 5β-androstanes – 226, pregnanes - 189, 5α-pregnanes – 743, 5β-pregnanes – 394 unique PubChem compound numbers. The complete set of results can be found in the provided Additional file 4. The assignment of the complete PubChem database took 30 minutes on a 4-core Linux system. We determined the precision of the assignment to the class “5β-androstanes” by manual inspection. Thus, 221 of the 226 assigned PubChem compounds were correctly assigned as 5β-androstanes, while 2 PubChem compounds were actually mixtures of compounds. For 3 of the 226 compounds it is arguable if they are to be considered 5β-androstanes as their substituents are so large that these substituents could be considered as scaffolds themselves. Therefore the annotation precision in this example is about 98%.

## Discussion

Chemical ontologies can be used for a variety of data and knowledge extraction and retrieval tasks. In the present work we have proposed and implemented design principles for a chemical ontology aiming at the automated annotation of compounds to their corresponding compound classes. To cover frequently used common class terms semi-automatically we have analysed the text corpus of all Medline abstracts using characteristic text chunks as described in the methods section. The most frequent class candidate terms were then manually defined in the chemical ontology via SMARTS expressions. This approach ensures an unbiased view on chemical compound classes and the high applicability of generated chemical ontology terms for text mining purposes. It also focuses the time consuming class definition effort on the most common class terms.

For the first time, we have implemented a structural definition of chemical classes by using combined sets of allowed and not allowed SMARTS rather than using a simple substructure definition as in previous approaches. In addition, allowed and not allowed SMARTS were connected by logical AND, OR and NOT operators to allow for a high precision, automated classification of chemical compounds.

**Figure 7:**
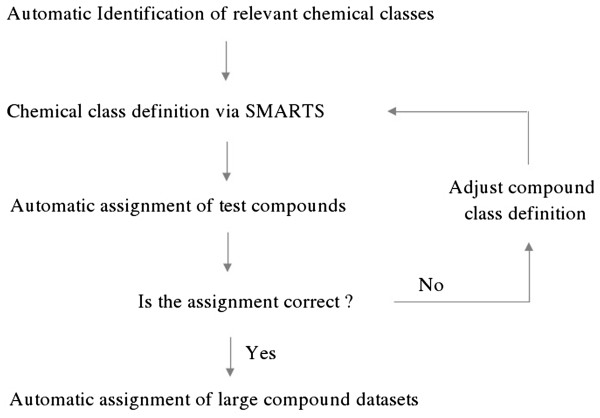
Process for chemical ontology construction.

To verify the applicability of the described approach we had to tackle several theoretical and technical problems. The implementation of the defined ontology construction rules was facilitated by the use of an especially developed chemistry ontology editor. In comparison to automated ontology generation approaches [18] we do not claim to construct a high precision chemical ontology in a self-organizing manner. However, also self-organizing ontology construction depends on the manual collection of compound training and test sets to compute chemical class definitions. We agree with the authors of [18] that a high precision chemical ontology can only be achieved in a semi-automated procedure. In our approach, we define chemical classes and their hierarchy or relations manually according to the process below: as shown in Figure 7 below.

With this approach we believe to cover more chemical class types as well as achieve higher precision of compound annotation results when compared to other chemical ontology generation procedures.

## Conclusions

Automated classification of compounds is possible using a hierarchy of chemical classes that are defined by a logical combination of SMARTS expressions. Our approach enables the annotation of structurally defined compounds in large chemical compound datasets as found in chemical databases or chemical structure-files with high performance. The annotation of chemical compounds was performed with high precision – the described ontology and approach is able to cover a wide range of different chemical class concepts with a resulting very precise annotation of compounds. While the ontology is expandable to any desired chemical class, one current limitation are poorly defined compounds such as polymers and metallo-organic compounds that cannot be described by SMARTS. However, this could potentially be improved by using appropriate chemical descriptions of such chemical entities as discussed above.

Also, it appears desirable to develop in the future a commonly accepted understanding of chemical classes, potentially leading to a standardization in forthcoming chemical ontologies. First proposals have been made in [11] for example on what is meant by broad and narrow compound class definitions, potentially also described by terms such as “derivatives”, “scaffolds” or “backbone”.

## Methods

### Identification of suitable compound classes

Chemical compound classes can be identified from a variety of sources. For example IUPAC nomenclature or chemistry text books for teaching chemistry are a good source. In addition to these, we have analysed Medline abstracts for chemical terms that are followed or preceded by expressions such as “analogue”, “derivatives”, “scaffold”, “skeleton”, “backbone”, or “substituted”. As a result we obtained a ranked list of 347,409 relevant chemical class terms which were regarded as chemical class candidates for ontology construction. The most frequent class candidate terms were “drugs” (574,369), “compounds” (469,069), “lipid” (334,920), “amino acid” (242,777) and so on – a more complete list is provided as Additional file 5.

### Chemistry ontology editor

To aid ontology development a chemical ontology editor (SODIAC - Structure Ontology Development and Individual Assignment Center) was developed. This editor allows building, editing and validating a growing chemical ontology using the SMARTS and SMILES rules described above for the definition of compound classes. As in other ontology editors, one may create, delete, edit, merge and move ontology nodes and also add definitions, synonyms, custom tags, external references and further data to each ontology class. SODIAC implements chemistry aware reasoning and allows drawing structures with a chemical structure editor. The handling of chemistry is based on ChemAxon's Java libraries [23]. Finally, the editor was used to annotate chemical compounds in the JChem database with their respective chemical compound classes. The compounds and their parent classes were subsequently loaded into a dictionary for the annotation of chemistry named entities in text with chemical ontology information.

### Annotation of PubMed

A database of compounds from PubChem was constructed from the SMILES of the respective compounds. The compound structures were converted to the respective isomeric SMILES and a resulting InChI [23] was calculated from their SMILES representation. Duplicate structures and their synonyms were joined based on the InChI identity of compounds. Compounds were loaded into a JChem database [23]. PubMed abstracts were annotated using an UIMA pipeline [27] and various dictionaries derived from ontologies in OBO format such as the chemistry ontology presented here. These annotated abstracts are accessible on a web-browser [28] for demonstration purposes. Finally, the resulting database of compounds was annotated using ontologies A.obo and B.obo by using the JChem Base [23] search functionality.

## Competing interests

All authors are employees of OntoChem GmbH, an organization that sells software and services in the field of bio- and chemoinformatics.

## Authors’ contributions

LW initiated and led the chemical ontology project, CB constructed the ontology, AP developed the chemical ontology editor, UL created the chemical database, TB created the UIMA pipeline and annotated PubMed. All co-authors contributed to the manuscript. All authors read and approved the final manuscript.

## Supplementary Material

Additional file 1 **A.obo - chemical ontology describing the ether class.** This ontology file contains the class definitions as described in this work to classify ethers in the OBO format, the oc_smarts tags contain the respective class SMARTS definitions. Click here for file

Additional file 2 **B.obo - chemical ontology describing the androstane and pregnane classes.** This ontology file contains the class definitions as described in this work to classify androstanes or pregnanes in the OBO format, the oc_smarts tags contain the respective class SMARTS definitions. Click here for file

Additional file 3 **Assignment precision.** This file contains the results of annotating a test set of 198 compounds from [18] with our complete ontology. Click here for file

Additional file 4 **Annotation results of androstane and pregnane compound classes in PubChem.** The complete set of 28,683,050 million compounds from the PubChem database was annotated with ontology B. The file contains in columns the ID number, the chemical class name, the number of assigned PubChem compounds, the respective PubChemID (CID), the corresponding SMILES, the correctness of assignment and a comment. Click here for file

Additional file 5 **Frequency of chemistry class terms in Medline.** This file contains the results of searching Medline abstracts for pre- or suffixes of the words “analogue”, “derivatives”, “scaffold”, “skeleton”, “backbone”, or “substituted”. Finally, frequencies of the unique terms were counted. Click here for file
